# The structural biology of oestrogen metabolism

**DOI:** 10.1016/j.jsbmb.2012.12.014

**Published:** 2013-09

**Authors:** Mark P. Thomas, Barry V.L. Potter

**Affiliations:** Department of Pharmacy & Pharmacology, University of Bath, Claverton Down, Bath, BA2 7AY, UK

**Keywords:** 17β-HSD, 17β-hydroxysteroid dehydrogenase, COMT, catechol-*O*-methyl transferase, DHEA(S), dehydroepiandrosterone (sulfate), DHETNA, O5′-[9-(3,17β-dihydroxy-1,3,5(10)-estratrien-16β-yl)-nonanoyl]adenosine, DNC, 3,5-dinitrocatechol, E1(S), estrone (sulfate), E2(S), estradiol (sulfate), E3, estriol, E4, estetrol, ER, estrogen receptor, E2B, 3-(((8*R*,9*S*,13*S*,14*S*,16*R*,17*S*)-3,17-dihydroxy-13-methyl-7,8,9,11,12,13,14,15,16,17-decahydro-6*H*-cyclopenta[*a*]phenanthren-16-yl)methyl)benzamide, FAD/FMN, flavin adenine dinucleotide/flavin mononucleotide, FG, formylglycine, HFG(S), hydroxyformylglycine (sulfate), mb-COMT, membrane-bound COMT, NADP^+^, nicotinamide adenine dinucleotide phosphate (oxidised), NADPH, nicotinamide adenine dinucleotide phosphate (reduced), PAP, 3′-phosphoadenosine-5′-phosphate, PAPS, 3′-phosphoadenosine-5′-phosphosulfate, s-COMT, soluble COMT, SAM, S-adenosyl methionine, SDR, short-chain dehydrogenase/reductase, Oestrogen, Protein structure, Reaction mechanism, Aromatase, Sulfatase, Sulfotransferase, 17β-Hydroxysteroid dehydrogenase

## Abstract

Many enzymes catalyse reactions that have an oestrogen as a substrate and/or a product. The reactions catalysed include aromatisation, oxidation, reduction, sulfonation, desulfonation, hydroxylation and methoxylation. The enzymes that catalyse these reactions must all recognise and bind oestrogen but, despite this, they have diverse structures. This review looks at each of these enzymes in turn, describing the structure and discussing the mechanism of the catalysed reaction. Since oestrogen has a role in many disease states inhibition of the enzymes of oestrogen metabolism may have an impact on the state or progression of the disease and inhibitors of these enzymes are briefly discussed.

This article is part of a Special Issue entitled ‘CSR 2013’.

## Highlights

► The structures of oestrogen-metabolising enzymes are discussed and compared. ► Substrate recognition and reaction mechanisms of these enzymes are discussed. ► The impact of structure on inhibitor design is briefly mentioned.

## 1 Introduction

Estrogens have many roles in the body including, but not limited to, in reproduction and the menstrual cycle [1–5], many breast cancers [6], the development of osteoarthritis [7], the prevention of heart disease [8], neuroprotection during cerebral ischaemia [9] and in multiple sclerosis [10], appetite and eating behaviour [11], fat metabolism [12], schizophrenia [13], autoimmunity [14], and auditory and visual processing [15]. Estrogens exert their effects in several ways [16–18]. In the ‘classic genome response’ oestrogen binds to specific intracellular oestrogen receptors (ERα and ERβ) that, subsequent to oestrogen binding, dimerise and translocate to the nucleus, where they modulate the transcription of target genes that contain oestrogen-responsive elements in their promoters. However, these same oestrogen receptors have also been shown (a) to bind to other transcription factors thus influencing the expression of genes that do not contain oestrogen-responsive elements in their promoters, and (b) to engage signal transduction pathways (that may include, but are not limited to, the activation of protein kinases), thus modulating cellular responses to oestrogen. Signal transduction pathways can also be activated by oestrogen binding to cell surface membrane bound receptors [16–18].

**Fig. 1:**
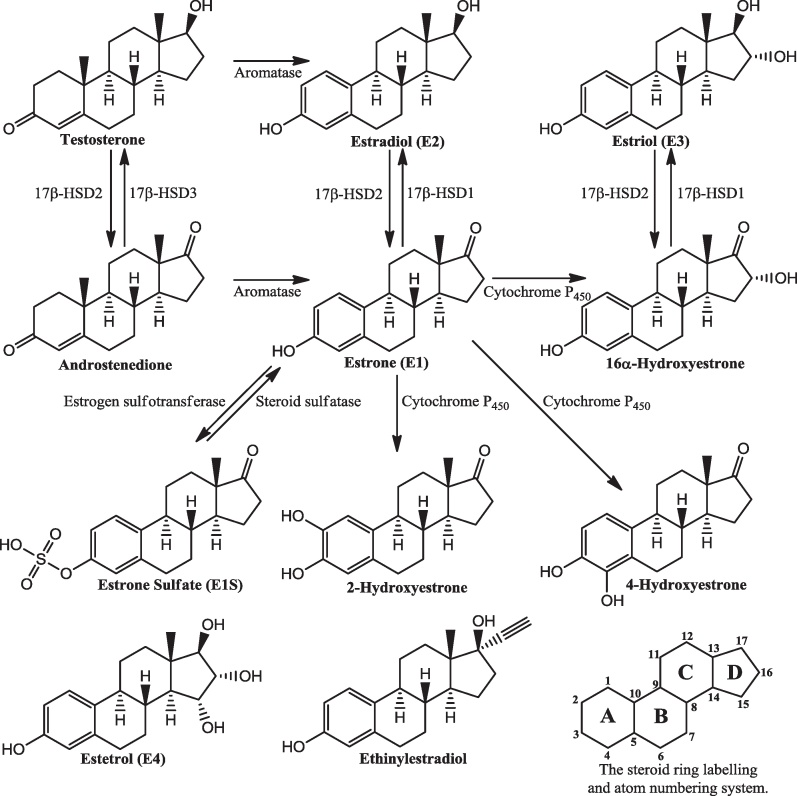
Pathways of oestrogen metabolism. Not all pathways are shown. There are tissue-specific variations in synthetic pathways: see [19]. The A-ring hydroxylated compounds are converted to the methoxy compounds by catechol *O*-methyltransferases. The structures of estetrol and ethinylestradiol are shown, as is the steroid ring labelling and atom numbering system.

The three most common estrogens are estrone (E1), estradiol (E2) and estriol (E3). A fourth oestrogen is estetrol (E4). Estradiol is the most potent. Estrone and estradiol are synthesised by the aromatisation of androstenedione and testosterone, respectively (Fig. 1). They can also be interconverted by the action of 17β-hydroxysteroid dehydrogenases (17β-HSDs). Estriol is synthesised from estrone via a 16α-hydroxyestrone intermediate. Although, in some tissues, estrogens can be made on demand, oestrogen can be stored in the form of estrone sulfate. This is synthesised from estrone by the action of oestrogen sulfotransferase with estrone being regenerated by the steroid sulfatase-catalysed hydrolysis of estrone sulfate. A review of many aspects of human steroidogenesis is available [19]. Estrogens are eliminated from the body mainly as the sulfated and glucuronidated derivatives [20, and references therein]. The first step in synthesising these conjugates is the generation of the hydroxylated derivatives. Hydroxylation occurs primarily at the 2-, 4- and 16-positions. The hydroxyl group can then be sulfated, glucuronidated or methylated.

Because there is little published work on estetrol it is discussed only briefly here. It is synthesised in the foetal liver, but its function is presently unknown. Several possible biosynthetic routes have been proposed starting from various androgens, estrogens, and their 3-sulfated derivatives, with the hydroxylations occurring in various different orders and, in the case of the androgens, the aromatisation occurring either before or after the hydroxylations. Evidence suggests that estetrol is made through multiple biosynthetic routes. See [21–29] for details. Estetrol will not be discussed further herein.

Given the role of estrogens in preventing, causing and exacerbating disease a good knowledge of how estrogens are synthesised and metabolised may help in the understanding and treatment of disease. This review looks in turn at each of the enzymes involved in oestrogen metabolism in terms of their structure and reaction mechanism, before a final section compares the various enzymes. Inhibition of the enzymes of oestrogen metabolism impacts on the amount of oestrogen in the body and this has been the subject of multiple therapeutic approaches leading to clinically active agents [30,31]. Also, synthetic estrogens, *e.g.*, ethinylestradiol (Fig. 1), have been widely employed in hormone replacement therapy [32] and, combined with a progestin, contraception [33,34]. Although inhibitors of these enzymes and synthetic estrogens are not the focus of this review they are briefly discussed, mainly through references to recent reviews. Unless otherwise stated, all discussions below refer to the human enzymes.

## 2 Methods

All dockings of ligands into binding sites were carried out using the GOLD software running in Gold Suite v5.0.1. All other manipulations of molecules, including building ligands and preparing proteins for docking runs, were carried out using the Schrödinger software running under Maestro 9.2.109. The protein crystal structures were downloaded from the PDB and run through the Protein Preparation Wizard. Hydrogens were added assuming a neutral pH and the resulting structures were put through a brief minimisation procedure. Visual inspection of these structures was used to identify those residues that form substrate binding sites. All figures of proteins and ligands were prepared using PyMOL (Delano Scientific). Figures of reaction schemes and molecules were prepared using ChemBioDraw Ultra 12.0. Multalin was used to perform sequence alignments (http://multalin.toulouse.inra.fr/multalin/multalin.html).

## 3 Oestrogen sulfotransferase

**Fig. 2:**
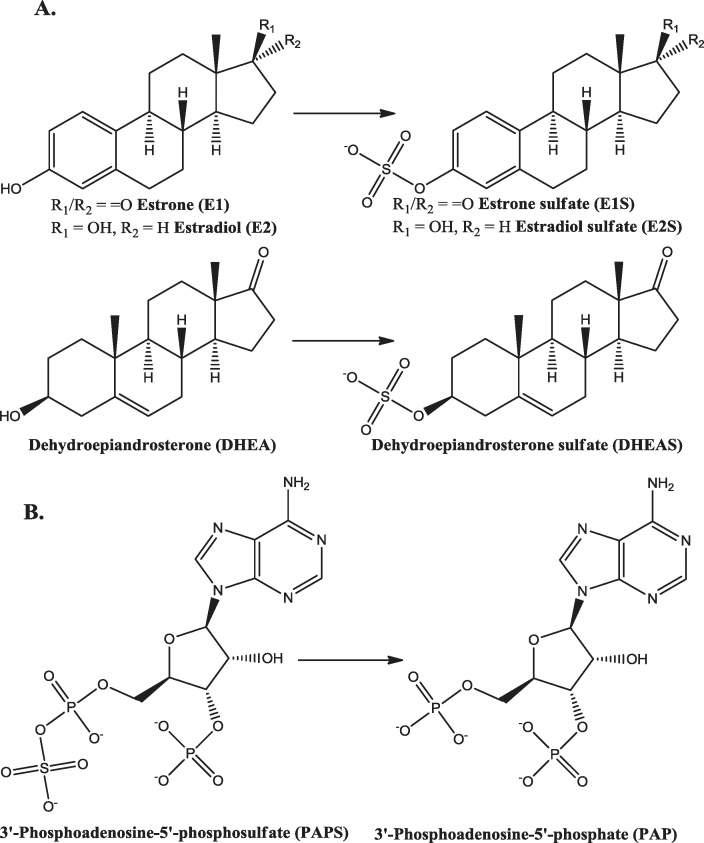
(A) The reactions catalysed by oestrogen sulfotransferase. (B) The cofactor, before and after reaction.

**Fig. 3:**
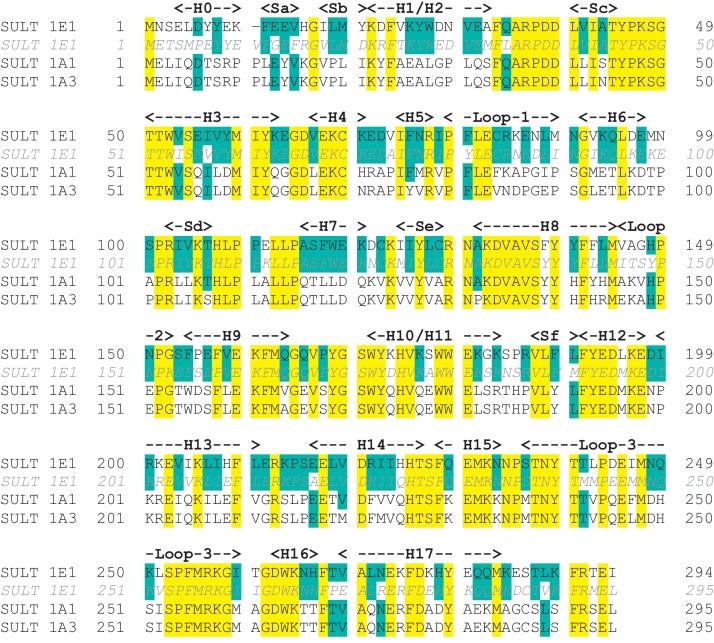
Sequences of sulfotransferases. Human SULT1E1 (oestrogen sulfotransferase) UniProt P49888. *Murine SULT1E1 (oestrogen sulfotransferase) UniProt*P49891. Human SULT1A1 (phenol sulfotransferase) UniProt P50225. Human SULT1A3 (phenol sulfotransferase) UniProt P50224. Residues identical across all four proteins are highlighted in yellow. Other residues identical to the human SULT 1E1 are highlighted in cyan. Secondary structural elements, as identified by PyMOL (Delano Scientific) in the 1G3M structure of human SULT1E1, are shown in bold: the numbering of these elements is consistent with that of [49]. H – helix, S – sheet.

Oestrogen sulfotransferase (EC 2.8.2.4, SULT1E1) is a cytosolic enzyme that catalyzes the sulfonation of estrogens, utilising 3′-phosphoadenosine-5′-phosphosulfate (PAPS) as the sulfate-donating cofactor [35,36] (Fig. 2). This enzyme has a high affinity for estrogens with maximal activity at about *K*_*m*_ = 20 nM [37]. There are two other sulfotransferases in the SULT1 class that catalyse the sulfonation of estrogens: SULT1A1 and SULT1A3 (both EC 2.8.2.1, phenol sulfotransferase). However, these enzymes have much lower affinities for estrogens, with maximal activity at about *K*_*m*_ = 25 μM. For all three human proteins the amino acid sequence is available, as is that of murine SULT1E1 (Fig. 3): SULT1A1 and SULT1A3 are 92.5% identical, and both are approximately 50% identical to SULT1E1. The sequence of murine SULT1E1 is 75% identical to that of the human protein.

**Fig. 4:**
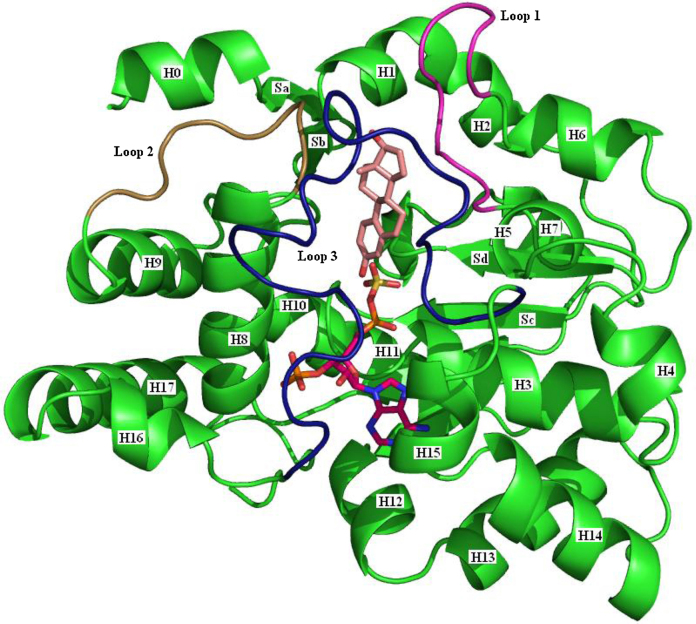
The three-dimensional structure of oestrogen sulfotransferase. The 1G3M (SULT1E1) structure [39] is shown. The cofactor PAPS (purple carbons) is taken from the 1HY3 structure [38], and the steroid (pink carbons) is from the 2D06 structure [44]. The helices, H0-H17 are identified, as are the sheets Sa-Sd. Sheets Se and Sf are hidden behind H3, H11 and H15. The flexible loops involved in binding the substrate and cofactor are identified thus: Loop 1, purple; Loop 2, brown; Loop 3, dark blue.

Available from the PDB are two crystal structures of human SULT1E1, three of murine SULT1E1, two of human SULT1A3, and eight of human SULT1A1 (Table 1). Given the high level of sequence identity it is not surprising that the three-dimensional structures are superimposable. The protein is a single domain globular structure (Fig. 4).

Thirty-one crystal structures of twelve different human sulfotransferases have been the subject of a recent review [48] that covers substrate specificity and mechanisms of action. An older review covering sulfotransferase structure and inhibitors is available [49].

**Fig. 5:**
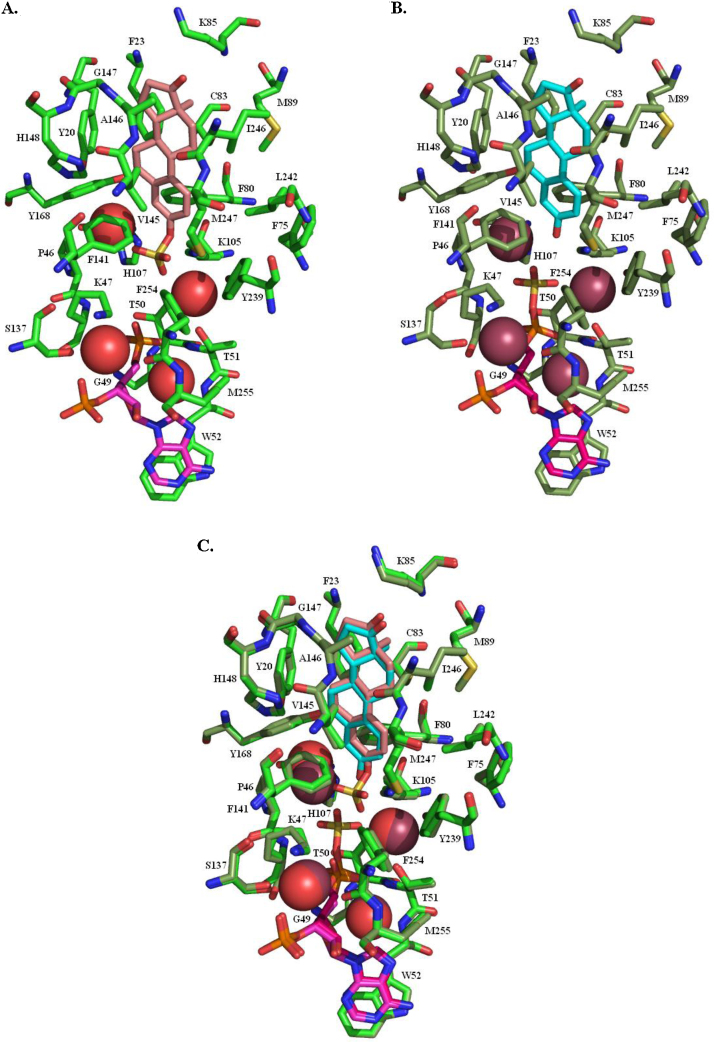
The binding site of oestrogen sulfotransferase. (A) Estrone sulfate (pink carbons) and 3′-phosphoadenosine-5′-phosphate (purple carbons) in the binding site. The red spheres are the oxygen atoms of water molecules in the binding site. (B) Estrone (cyan carbons) and 3′-phosphoadenosine-5′-phosphosulfate (purple carbons) in the binding site. The brown spheres are the oxygen atoms of water molecules in the binding site. (C) An overlay of A and B, showing the short distance the sulfate has to move during the reaction.

Fig. 5 shows the structure of the binding site of human SULT1E1. To generate Fig. 5A the reaction product was docked into the 1HY3 structure after the sulfate had been removed from the cofactor. To generate Fig. 5B the substrate was docked into the 1HY3 structure. In both instances, subsequent to docking, the protein ligand complex was put through 1000 rounds of minimisation prior to preparing the figures. A comparison of the two structures (Fig. 5C) shows that three of the water molecules have different positions but the ligand moves only slightly, and the surrounding residues barely move at all.

The substrate and cofactor binding site takes the form of a tube, open at both ends, running through the core of the protein. The residues forming the substrate binding site are predominantly hydrophobic and enclose the substrate quite closely. These residues (Y20, F23, P46, F75, F80, C83, K85, M89, K105, H107, F138, F141, V145, A146, G147, H148, Y168, Y239, L242, I246 and M247) are mainly from the three flexible loops (Fig. 4), loop 1 (between helices 5 and 6), loop 2 (between helices 8 and 9), and loop 3 (between helices 15 and 16), but also helices H1 and H8 and sheet Sd. A particularly close interaction is that of the hydrogens attached to C6 and C7 in the B-ring with the face of the Y20 ring. The side chain of F141 points directly at the A-ring of the substrate to have a face–edge π–π interaction. K105 has a hydrogen bond to the oxygen at the 3-position. These interactions position the substrate hydroxyl so that it can act as the acceptor in the sulfate transfer.

The cofactor is enclosed by nineteen residues (K47, S48, G49, T50, T51, W52, K105, H107, R129, S137, Y192, T226, F228, M231, F254, M255, R256, K257 and G258) that are predominantly in helices 3, 8 and 15, and loop 3. The PAPS adenine moiety has a face–face π–π stacking interaction with W52 and hydrogen bonds to Y192 and T226. The ribose-3-phosphate has hydrogen bond interactions with R129, K257, G258 and R256, and the 5-phosphate with G49 and T51. The sulfate is held in position by interaction with K47 and K105. There are four water molecules that may play a role in catalysis and/or that interact with the substrate or cofactor.

A comparison of all human sulfotransferase structures suggests that the binding of substrate and PAPS can occur in a random order, and that substrate binding may be cooperative with prior cofactor binding, but not *vice versa* [50]. It seems likely that loop 3 must move to enable both substrate and cofactor to bind. The ends of this loop (helices 15 and 16) are in the vicinity of the cofactor: if the entire loop opens as if the ends of the loop act as a hinge then the prior binding of the cofactor may position the remainder of the loop to more easily bind the substrate. This is supported by crystal structures of SULT1A1 in complex with PAP and substrate where the binding site is very similar to that observed when just PAP is bound [47]. (The conformation of this loop subsequent to PAPS binding is also thought to influence substrate binding in human SULT2A1 [51].) There are some reports that at high substrate concentrations sulfotransferases, particularly SULT1A1, suffer from substrate inhibition [46,50]. Whether this is of relevance *in vivo* is undetermined.

**Fig. 6:**
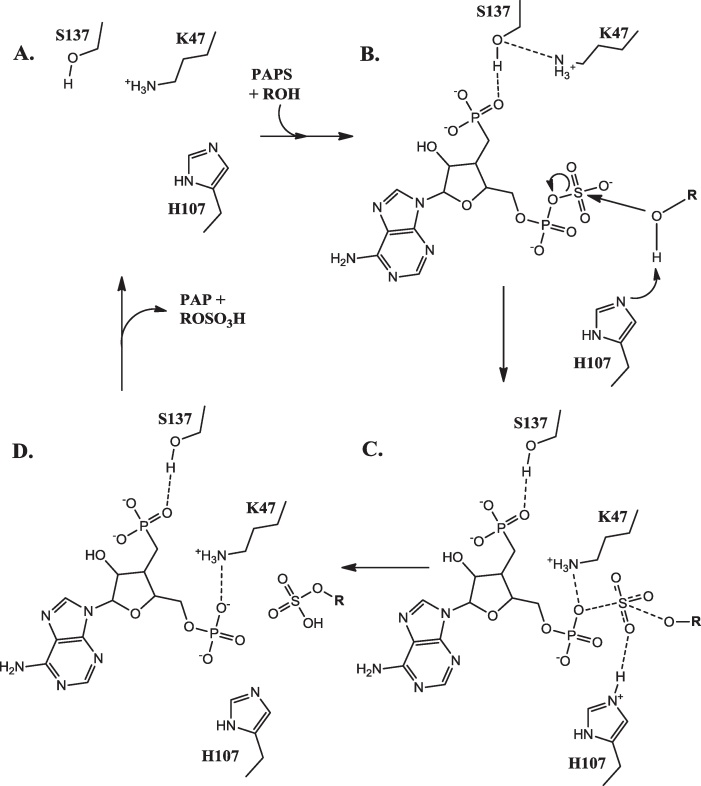
The oestrogen sulfotransferase reaction mechanism. (A) The apoenzyme. (B) The enzyme-PAPS–substrate complex. (C) The intermediate. (D) The enzyme–PAP–product complex.

Adapted from [52].

Three conserved residues, K47, H107 and S137, are thought to be involved in the reaction mechanism (Fig. 6) [38,52]. S137 forms a hydrogen bond to K47, thus preventing the side chain nitrogen of K47 from interacting with the bridging oxygen. This prevents PAPS hydrolysis. Upon substrate binding, the catalytic base H107 abstracts a proton from the substrate hydroxyl, thus enabling nucleophilic attack at the sulphur atom in PAPS. The consequent accumulation of partial negative charge on the bridging oxygen of PAPS disrupts the interaction between S137 and K47, thus enabling K47 to interact with the 5′-phosphate. This assists in the dissociation of the sulfyryl group and completes the catalytic reaction cycle [52,53]. The sulfonation takes place via an in-line displacement mechanism though whether the mechanism is associative (S_N_2-like) or dissociative (S_N_1-like) is not yet clear [53,54].

## 4 Steroid sulfatase

Steroid sulfatase (EC 3.1.6.2, aryl sulfatase C, steryl-sulfatase) catalyzes the conversion of estrone sulfate to estrone (Fig. 1), and dehydroepiandrosterone sulfate (DHEAS) to dehydroepiandrosterone (DHEA) (the reverse of the reaction shown in Fig. 2). The protein is found in the lumen of the endoplasmic reticulum and, in terms of tissue distribution, is probably ubiquitous at low levels, with greater amounts in those tissues associated with reproduction. The biology of steroid sulfatase has been reviewed [55] as has the role of steroid sulfatase in oestrogen metabolism [56].

**Fig. 7:**
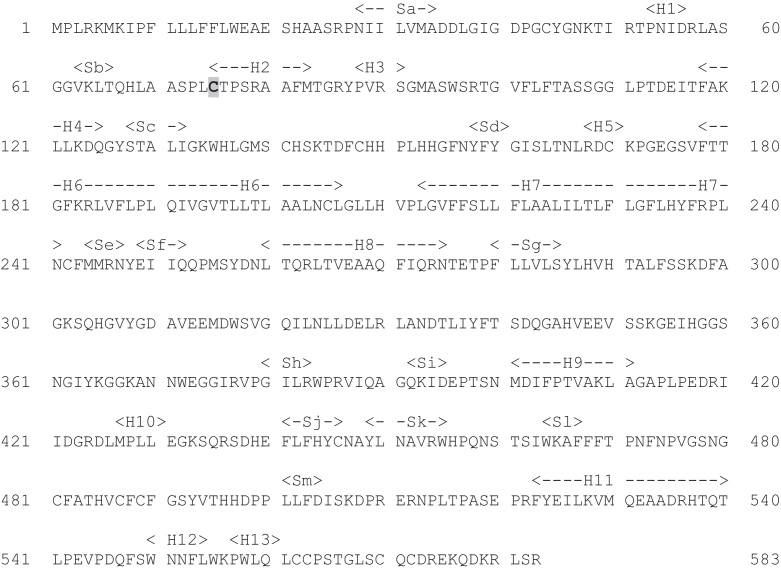
The sequence of steroid sulfatase (UniProt P08842). Residue C75 (highlighted) undergoes post-translational modification to formylglycine [60].

**Fig. 8:**
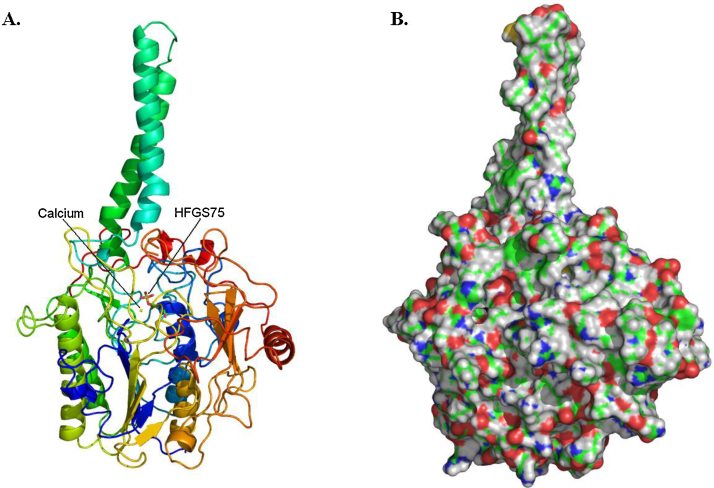
The three-dimensional structure of steroid sulfatase. PDB code 1P49[59]. (A) A cartoon of the structure coloured blue at the N-terminus through to red at the C-terminus. Residue HFGS75 and the calcium ion are in the middle of the structure. (B) The surface of the protein showing the hydrophobic nature of the membrane-embedded tail at the top.

**Fig. 9:**
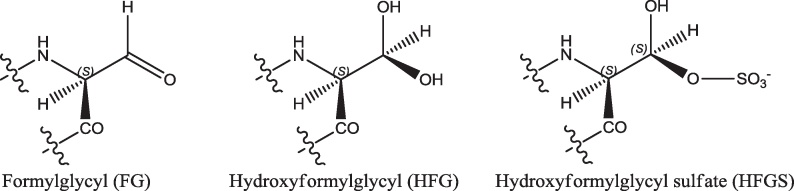
The structures of formylglycyl, hydroxyformylglycyl and hydroxyformylglycyl sulfate residues.

The gene has been cloned and sequenced, the protein expressed and purified [57,58] and shown to consist of 583 residues (Fig. 7). The structure of the protein has been solved (PDB code 1P49: [59]) and shown to have a globular head (approximately 65 Å × 50 Å × 45 Å) and a hydrophobic tail about 40 Å long (Fig. 8). This tail is embedded in the lumenal membrane of the endoplasmic reticulum. The crystal structure shows that several residues undergo post-translational glycosylation. Another post-translational modification is that of C75 to formylglycine (FG) (Fig. 9) [60]. This is further modified, by hydration, to form the *gem*-diol hydroxyformylglycine (HFG), though the resting state of the enzyme appears to be hydroxyformylglycine sulfate (HFGS) with the sulfate linked to the pro-(*S*) hydroxyl group.

**Fig. 10:**
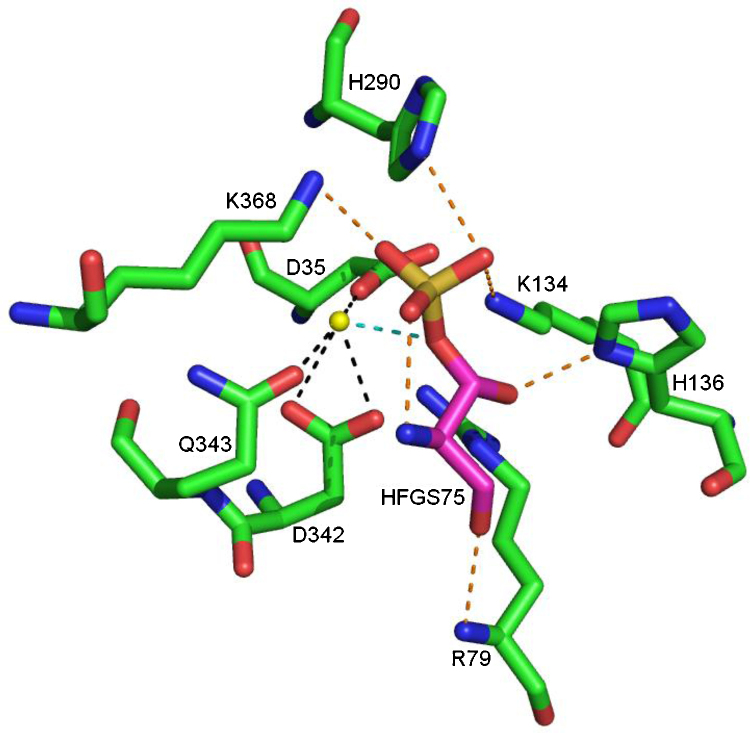
The interactions of the calcium ion and HFGS75 in the binding site of steroid sulfatase. Hydrogen bonds involving HFGS75 are shown in orange. Interactions with the calcium ion are shown in black, except for the interaction with HFGS75 which is shown in blue. Based on the 1P49 structure [59]. (For interpretation of the references to colour in this figure legend, the reader is referred to the web version of this article.)

Visual inspection of the structure shows that a number of hydrogen bonds help position the HFGS and, specifically, the side chain in the binding site (Fig. 10). The HFGS backbone carbonyl may be involved in a hydrogen bond with the backbone NH of R79, and the HFGS side chain hydroxyl may form a hydrogen bond to the side chain of K134 and H136. There are four or, possibly, five interactions of the sulfate with the protein that may serve to position the sulfate during the reaction. The first of these four is an internal hydrogen bond from the HFGS backbone NH, and the other four involve the side chains of H290, K134 and, possibly, K368. A calcium ion in the binding site has an interaction with one of the HFGS sulfate oxygens. The calcium ion also has charge–charge interactions with the side chains of D35, D36, D342 and Q343. The substrate binding site is formed by residues L74, HFGS75, R98, T99, G100, V101, L103, L164, T165, L167, R168, V177, F178, T180, G181, H290, T291, H346, E349, K368, N447, T484, H485, V486, F488 and C489.

**Fig. 11:**
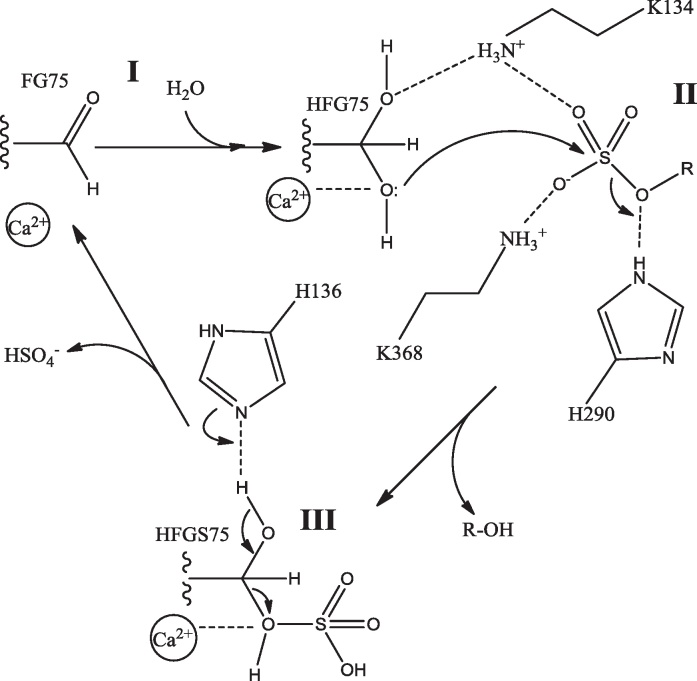
Proposed catalytic mechanism of steroid sulfatase.

Adapted from [61].

One proposed mechanism for the steroid sulfatase-catalysed reaction is shown in Fig. 11 [61]. In Step **I** FG75 is activated by a water molecule to form the *gem*-diol. In step **II** the substrate sulphur undergoes nucleophilic attack by the *pro*-(*S*) hydroxyl following its activation by Ca^2+^. This results in the sulfate moiety being covalently linked to FG75 and the unconjugated substrate being released with the sulfate being replaced by a hydrogen abstracted from H290. In step **III** the *pro*-(*R*) hydroxyl of HFGS75 is deprotonated by H136 and the ester bond is broken by attack of the activated free hydroxyl. The sulfate is released and FG75 is regenerated. Release of the sulfate may require an additional water molecule as a nucleophile. This water may accompany the incoming substrate, shielding the charged sulfate during its passage into the substrate binding site.

**Fig. 12:**
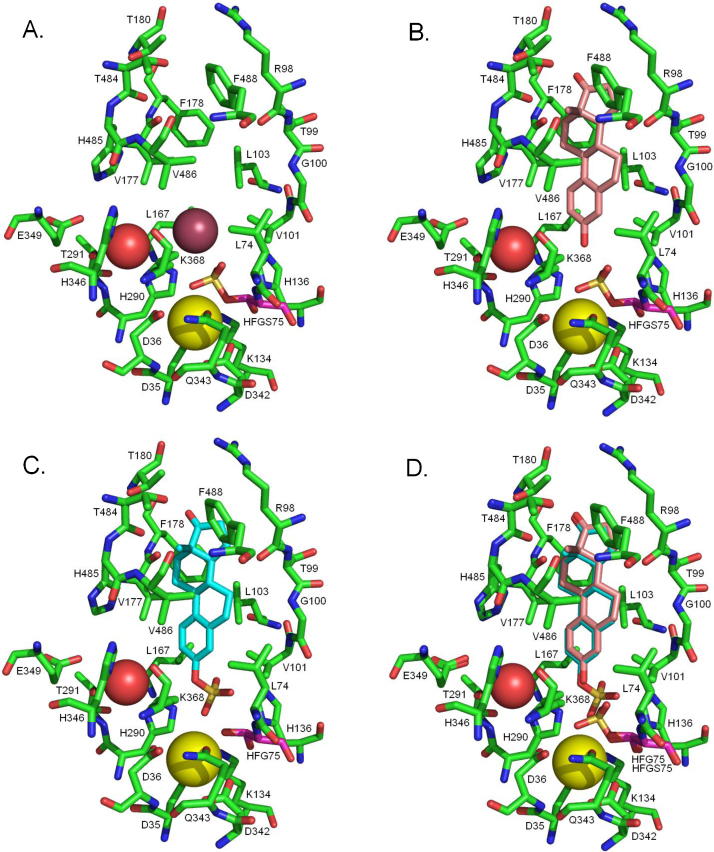
The steroid sulfatase binding site. (A) The crystal structure of the steroid sulfatase substrate binding site. HFGS75 is shown with purple carbons. The calcium ion is the yellow sphere. The putative catalytic water is the red sphere. The brown sphere is the only other water present in the substrate binding site in the crystal structure. For both water molecules only the oxygen is shown. (B) Estrone docked into the substrate binding site of steroid sulfatase. The water in the substrate binding site was removed prior to docking: it occupies the space taken by the estrone hydroxyl. (C) Estrone sulfate docked into the substrate binding site of steroid sulfatase. Prior to docking the sulfate was removed from residue 75 to leave hydroxyformylglycine. (D) An overlay of B and C showing the identical docked pose of the estrone core and how little the sulfate has to move during the reaction. (For interpretation of the references to colour in this figure legend, the reader is referred to the web version of this article.)

The reaction site lies at the base of a long narrow pocket that opens beside the hydrophobic tail. This suggests that the substrate and product may have to travel through the endoplasmic reticulum membrane in order to enter and leave the substrate binding site. The binding site itself is formed predominantly from hydrophobic residues (Fig. 12). The crystal structure has two water molecules in the substrate binding site, one of them putatively being the catalytic water, but the other being a crystallographic water interacting with the sulfate of HFGS75. This second water has to be removed prior to performing docking experiments to determine how the substrate and product might fit into the binding site.

Inhibition of steroid sulfatase (together with the inhibition of aromatase – see below) should reduce the amount of oestrogen in the body, particularly in target tumour tissue, and be beneficial in a range of hormone-dependent diseases. The development of steroid sulfatase inhibitors has been the subject of a number of recent reviews [62–66].

## 5 17β-Hydroxysteroid dehydrogenases

There are fourteen different vertebrate enzymes classified as 17β-hydroxysteroid dehydrogenases of which twelve have been found in human tissues [67]. Despite their name the preferred substrate of some of these enzymes is other than steroids and, when the substrate is a steroid, the reaction may be either an oxidation or a reduction depending on the cofactor and cellular localisation [68]. 17β-Hydroxysteroid dehydrogenase type 1 (EC 1.1.1.62, 17β-HSD1) catalyses the conversion of estrone to estradiol, and of 16α-hydroxyestrone to estriol (Fig. 1). The reverse reactions are catalysed by 17β-HSD2 which also facilitates the conversion of testosterone to androstenedione, the reverse reaction of which is catalysed by 17β-HSD3.

**Fig. 13:**
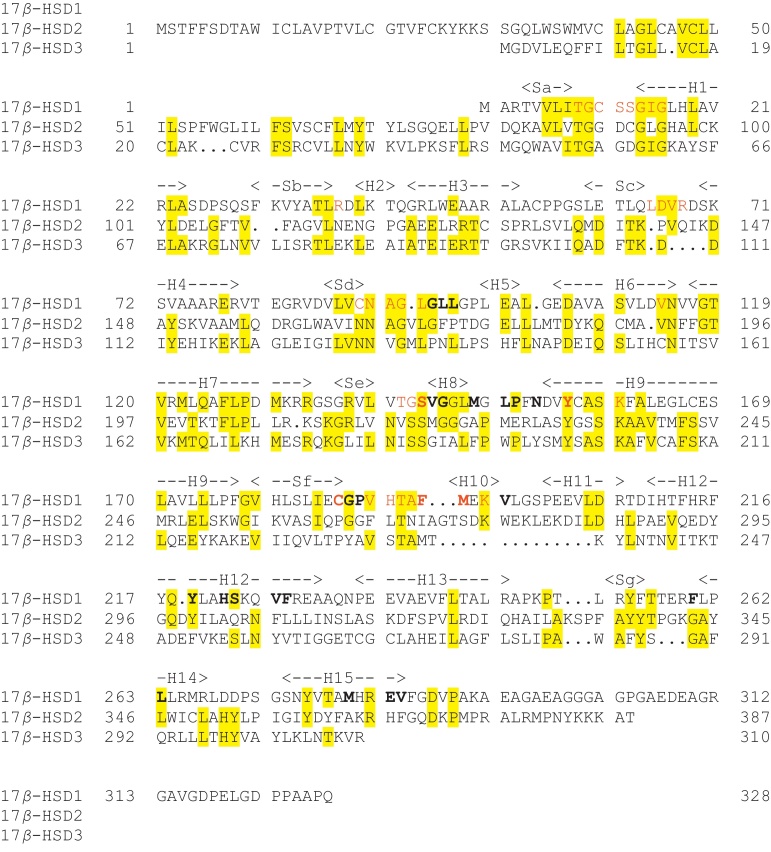
Sequence alignment of 17β-hydroxysteroid dehydrogenases. An alignment of the 17β-HSD1 (UniProt ID code P14061), 17β-HSD2 (UniProt ID code P37059) and 17β-HSD3 (UniProt ID code P37058) sequences. Identical residues are highlighted in yellow. Those residues in 17β-HSD1 forming the substrate binding site, as identified by visual inspection of the structure, are shown in bold, and those forming the NADPH binding site are shown in red. Secondary structure elements in 17β-HSD1 are identified as H – helix and S – sheet. (For interpretation of the references to colour in this figure legend, the reader is referred to the web version of this article.)

**Fig. 14:**
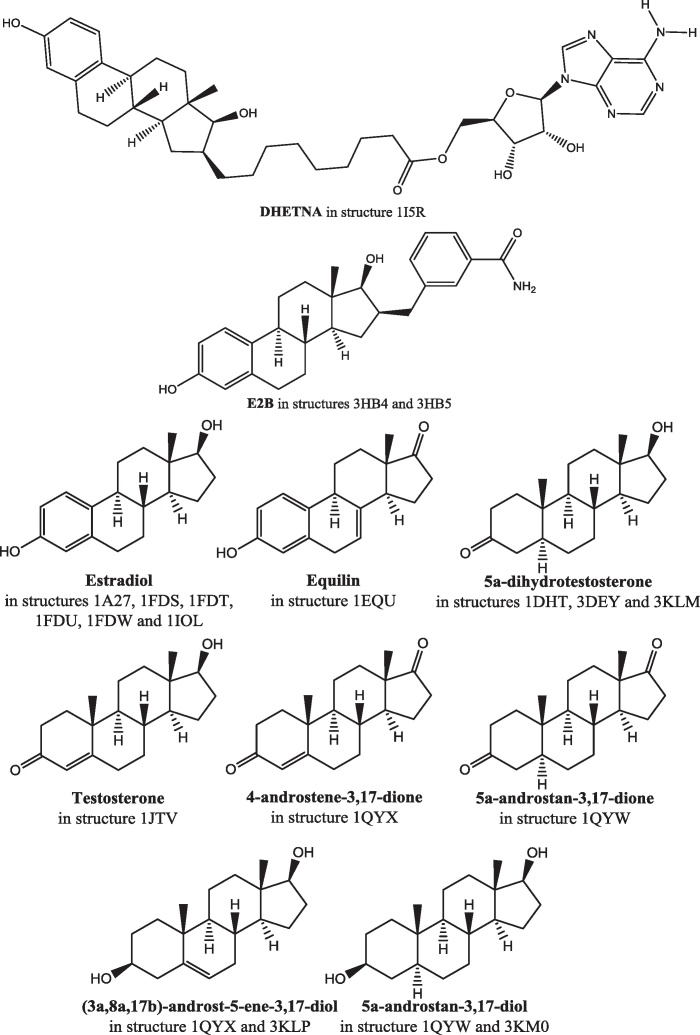
The structures of the ligands in the crystal structures of 17β-HSD1. DHETNA: O5′-[9-(3,17β-dihydroxy-1,3,5(10)-estratrien-16β-yl)-nonanoyl]adenosine. E2B: 3-(((8*R*,9*S*,13*S*,14*S*,16*R*,17*S*)-3,17-dihydroxy-13-methyl-7,8,9,11,12,13,14,15,16,17-decahydro-6*H*-cyclopenta[*a*]phenanthren-16-yl)methyl)benzamide.

**Fig. 15:**
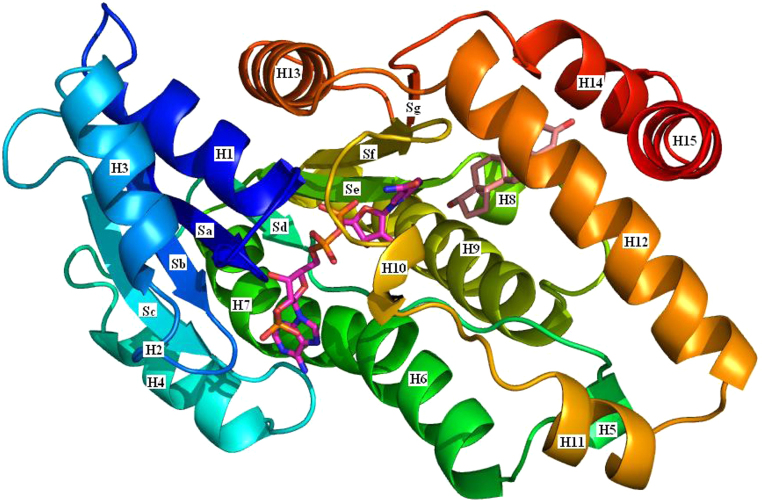
The structure of 17β-HSD1. A cartoon of the 17β-HSD1 structure coloured blue at the N-terminus through to red at the C-terminus. The secondary structure elements are labelled as in Fig. 13: H – helix and S – sheet. NADPH is shown with purple carbons and estradiol with pink carbons (based on the 1A27 structure [69]). (For interpretation of the references to colour in this figure legend, the reader is referred to the web version of this article.)

All three enzymes are members of the short-chain dehydrogenase/reductase (SDR) structural family. The aligned sequences are shown in Fig. 13. Listed in Table 2 are the twenty-two publicly available structures of 17β-HSD1. Most of these structures have a ligand in the substrate binding site: the structures of these ligands are shown in Fig. 14. The gene can be translated into 328 amino acids but the mature protein has lost the initiating methionine and, for historical reasons, is often (but not always) numbered A1–Q327. Herein the A1–Q327 numbering is used. There are no publicly available crystal structures of either 17β-HSD2 or 17β-HSD3, but the overall 3D structure of both is believed to be similar to that of 17β-HSD1 (Fig. 15).

The protein is built around a core of seven parallel β-strands with at least one helix between successive strands. This makes up the Rossman fold typical of nucleotide binding proteins. The loop between sheet 1 and helix 1 has the GxxxGxG motif (residues G9–G15, where G is glycine and x any other residue) that is common to oxidation/reduction enzymes that bind nicotinamide cofactors. S142, at the end of sheet 5, and Y155 and K159, in helix 9, have been shown to be essential for activity [81].

The substrate binding site is formed by residues G94, L95, L96, S142, V143, G144, M147, L149, P150, N152, Y155, C185, G186, P187, F192, M193, V196, Y218, H221, S222, V225, F226, F259, L262, M279, E282 and V283. Of these twenty-six residues only fourteen are in secondary structural elements, *i.e.*, helices or sheets, with the remainder being in loops between the structural elements. One of these loops, the substrate binding loop comprising residues H189–V196, is flexible enough that it is observed in two conformations in the 1FDT structure. This loop probably has to move to allow the substrate into the binding site. The following residues form the NADPH binding site: T8, G9, C10, S11, S12, G13, I14, G15, R37, L64, D65, V66, R67, C89, N90, A91, G92, L93, V113, T140, G141, S142, Y155, K159, C185, V188, H189, T190, A191, F192, M193 and K195.

**Fig. 16:**
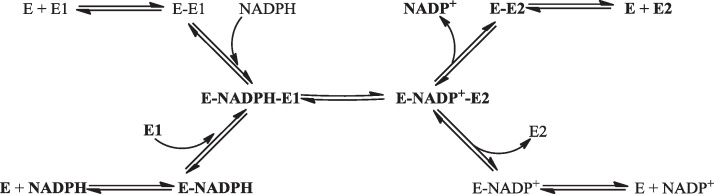
The proposed random order bi–bi mechanism of 17β-HSD1. The mechanism believed to be preferred is shown in bold [83,84]. E – enzyme, E1 – estrone, E2 – estradiol.

**Fig. 17:**
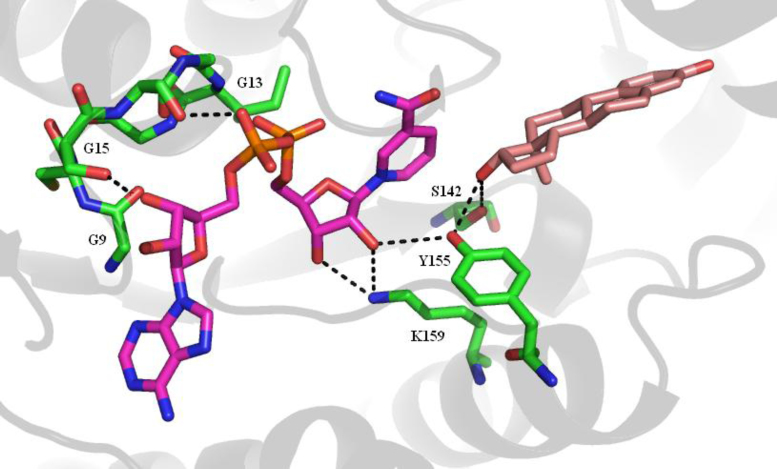
The substrate binding site of 17β-HSD1. The nucleotide binding GxxxGxG motif and the residues involved in catalysis are shown in green. The NADPH is in purple and the estradiol in pink. Secondary structure elements are shown in grey. Some of the possible hydrogen bonds are shown by the black dashed lines. (For interpretation of the references to colour in this figure legend, the reader is referred to the web version of this article.)

The hydride transfer reactions catalysed by these enzymes are intrinsically reversible but, *in vivo*, are effectively unidirectional due to the relative concentrations of NADPH and NADP^+^ [82]. Based on the measurement of isotope exchange rates between substrate-product pairs the reaction catalysed by 17β-HSD1 has been reported to be a random order bi–bi mechanism [83] (Fig. 16). However, a dynamics study based on crystal structures of the apo enzyme and binary and ternary complexes has suggested that there is a preferred order with NADPH binding before the substrate, and the NADP^+^ being released before the product [84]. In the reaction the *pro*-(*S*) hydride is transferred from NADPH to the alpha face of the estrone C17. A hydride is then transferred to the C17 oxygen, stabilised by interaction with the hydroxyl of S142, from the hydroxyl of Y155. There is then postulated to be a hydride transfer network involving the NADPH ribose hydroxyl, the K159 amino moiety and a water molecule that results in the formation of a hydronium ion [84] (Fig. 17). Work with the 17β-HSD from the filamentous fungus *Cochliobolus lunatus* has shown that cofactor dissociation is the slowest step of the reaction and that the catalytic activity might be modulated by differences in the conformation of the substrate binding loop consequent upon cofactor and substrate binding [85]. Enzymes of the SDR family are typically active as dimers or tetramers, and work with the *C. lunatus* enzyme suggests that dimerisation may be necessary for activity [86].

Of the estrogens estradiol is the most potent and the inhibition of estradiol production is beneficial in the treatment of oestrogen-dependent diseases. Inhibition of 17β-HSD1 should prevent estradiol production, though the concomitant inhibition of aromatase (see below) and steroid sulfatase is probably necessary for greater reduction of estradiol levels. A variety of steroidal and non-steroidal inhibitors have been synthesised that have IC_50_ values in the low nanomolar range. For recent reviews of these enzymes and their inhibitors see [87–89].

## 6 Aromatase (cytochrome P450 19A1, oestrogen synthase)

**Fig. 18:**
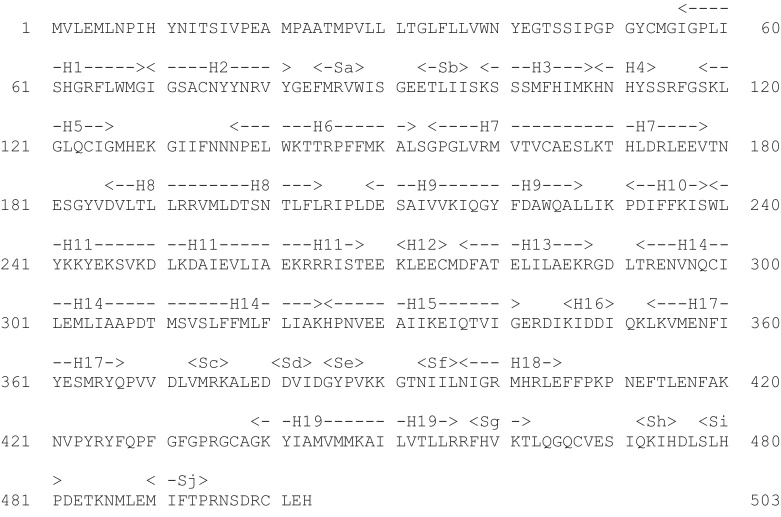
The sequence of aromatase (UniProt ID code P11511). H – helix and S – sheet.

**Fig. 19:**
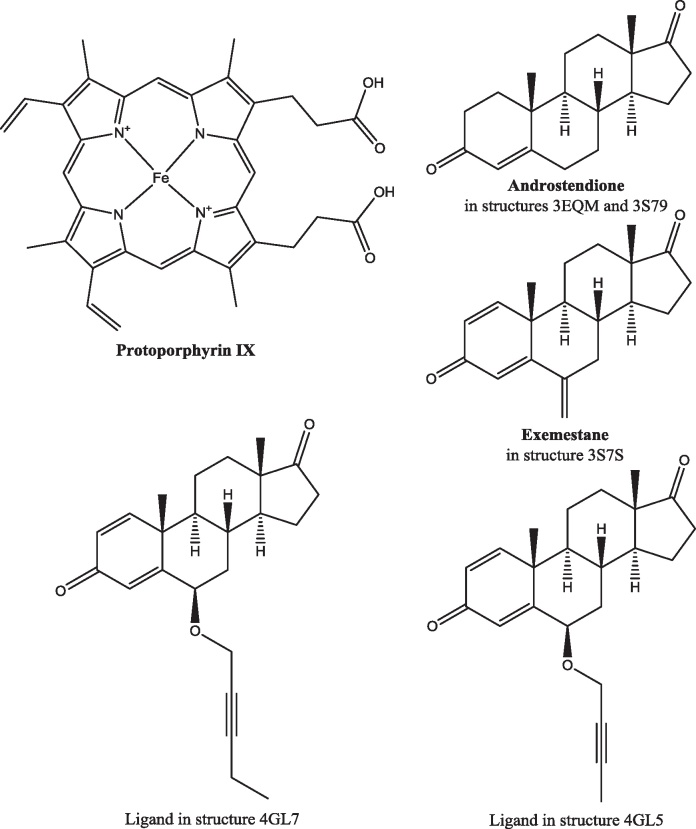
The structure of ligands in the aromatase binding site. The protoporphyrin is in all the crystal structures of aromatase.

Aromatase (cytochrome P450 19A1, cytochrome P450AROM, oestrogen synthase) catalyzes the aromatisation of the ‘A’ ring of androstendione to produce estrone and the aromatisation of the ‘A’ ring of testosterone to produce estradiol (Fig. 1). The enzyme consists of 503 amino acids (Fig. 18) and an iron-containing haem group (protoporphyrin IX; Fig. 19). The protein is glycosylated on N12 [90]. Other post-translational modifications are performed by the tyrosine kinases c-Src [91] and PTP1B [92]: phosphorylation of Y361 by c-Src increases aromatase activity. The enzyme is found in the cytoplasm bound to the endoplasmic reticulum with the *N*-terminal residues probably penetrating through the membrane and the glycosylated residue in the lumen of the endoplasmic reticulum. However, it has been suggested that the *N*-terminal residues are required not so much for membrane association as for orientation [93]. There is evidence to suggest that aromatase forms dimers or higher oligomers [93,94]. The proposed mode of oligomerisation may be a further means of regulating enzyme activity as it would prevent the phosphorylation of Y361 [94].

**Fig. 20:**
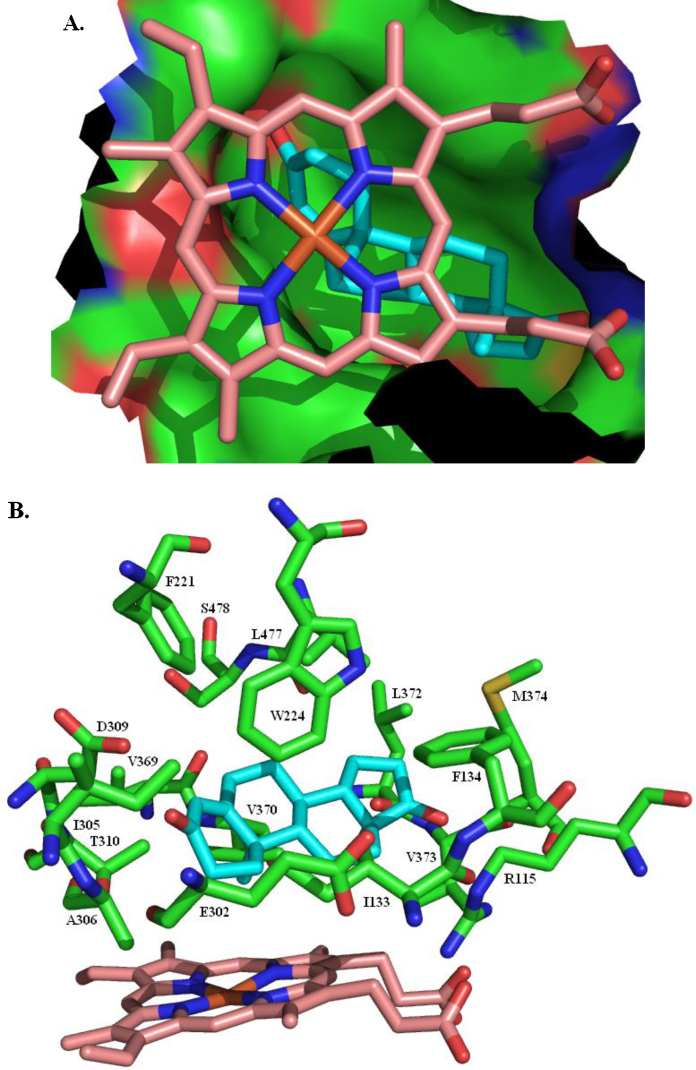
The aromatase substrate binding site. (A) The haem-capped cavity tightly enclosing androstenedione. (B) The residues forming the substrate binding site.

There are five publicly available crystal structures of human placental aromatase: PDB code 3EQM [95,96] has androstenedione in the substrate binding site, as does 3S79 [97]; the inhibitor exemestane (Fig. 19) is in the 3S7S structure [97]; the 4GL5 and 4GL7 structures [97] both have *O*-linked alkyne chains attached to the 6-position of the steroid ring. All five structures are of residues S45–N496. They reveal a predominantly helical single domain protein with the ligand tightly enclosed in a largely hydrophobic pocket, situated deep within the protein, that is formed by residues R115, I133, F134, F221, W224, E302, I305, A306, D309, T310, V369, V370, L372, V373, M374, L477 and S478, and the haem (Fig. 20). There is a hydrogen bond between the backbone NH of M374 and the ligand carbonyl at the 17-position and another between the side chain of D309 and the ligand carbonyl at the 3-position. In the 3EQM and 3S79 structures the androstenedione has an identical pose, as do the haems and the residues forming the binding site. The B, C and D rings of exemestane overlay the equivalent rings in androstenedione but, because of the additional double bond in the A ring of exemestane, the A ring is slightly distorted relative to the androstenedione A ring. The C6-methylidene carbon is accommodated by slight shifts (less than 1 Å) in the position of T310 and, to a lesser extent, S478. Despite the greater size of the 6-substituent in the 4GL5 and 4GL7 structures there is no further significant movement of any of the amino acid residues in the substrate binding site.

**Fig. 21:**
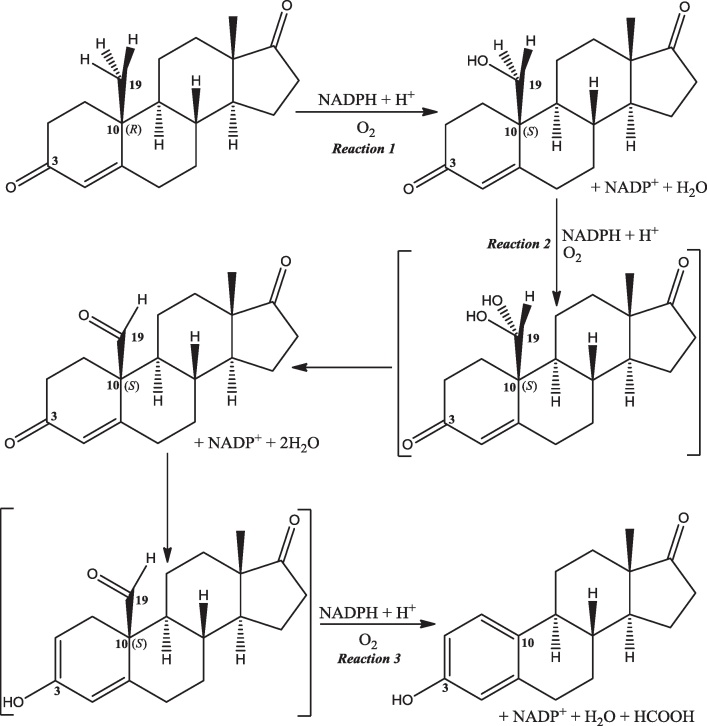
The mechanism of the aromatase-catalysed reaction. Carbons 3, 10 and 19 are identified as is the stereochemistry of C10.

The aromatisation occurs in a three-step mechanism [98,99] (Fig. 21). Two consecutive hydroxylations generate, first, a hydroxyl at C19 and, second, a *gem*-diol intermediate that dehydrates to leave an aldehyde [100]. The ketone at the 3-position then enolises and the aldehyde is cleaved from C10 and ejected as methanoic (formic) acid [101]. This implies that the aromatase catalytic centre can catalyse two different types of reaction (Eqs. (1) and (2)). Note that the first hydroxylation of C19, whilst leaving the absolute configuration of C10 unchanged, does change the stereochemistry of C10 from *R* to *S*.(1)
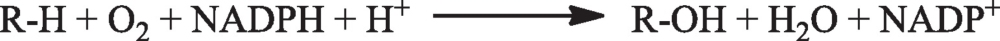
(2)
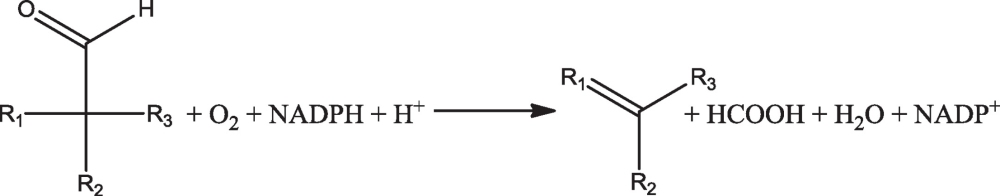


Residues involved in the catalytic act probably include A306, D309 and T310. The binding site opens to the ER membrane so the substrate has to traverse the membrane in order to enter the binding site. NADPH does not bind directly to aromatase. Two electrons are transferred from NADPH to the aromatase haem group *via* NADPH cytochrome P450 reductase, an FAD/FMN-containing protein [102,103].

Atomic force microscopy has been used to observe aromatase dimers in membranes, and possibly higher order interactions [93]. Computational modelling of aromatase in membranes suggests that a dynamic quaternary organisation together with fluctuations of the active site, the cavity enclosing the haem and the substrate access channel are necessary for activity [104,105].

**Fig. 22:**
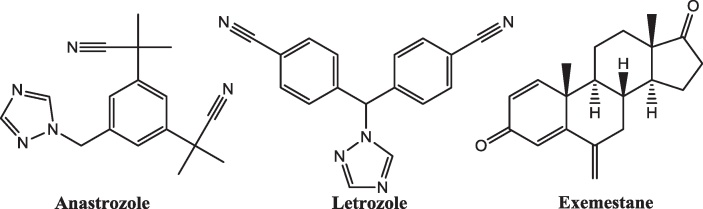
The structure of some clinically used aromatase inhibitors.

The inhibition of aromatase is a suitable treatment for a number of clinical conditions that are caused or aggravated by the overproduction of oestrogen. Aromatase inhibitors such as anastrozole, letrozole and exemestane (Fig. 22) have found roles in the treatment of breast cancer [106], ovarian cancer [107] and growth maximisation in puberty in children with short stature [108], and it has been suggested that aromatase inhibitors may have a role to play in treating some cases of lung cancer [109].

## 7 Enzymes of steroid hydroxylation

Aromatase, cytochrome P450 19A1, catalyzes the hydroxylation of testosterone and androstenedione at C19 prior to aromatising the A-ring. Other cytochrome P450s catalyse hydroxylations at other sites [110], most commonly the 2-, 4- and 16-positions. It has been argued that which cytochrome is involved with which hydroxylation is dependent on both the body tissue and the substrate concentration [111].

Cytochrome P450s 1A1, 2C19, 3A4 and 3A5 are amongst those reported to catalyse hydroxylation at the 16α-position [111,112]. This generates 16α-hydroxyestrone which can then be converted to estriol by 17β-HSD1. Reported to be involved in both 2- and 4-hydroxylations are cytochrome P450s 1A1, 1A2 and 1B1, though the relative importance of these might depend upon the tissue, and others are probably involved [111]. In the PDB are the structures of more than 500 cytochrome P450s, over 100 of which are of human proteins. The structures of CYPs 1A2, 1B1 and 3A4 are briefly discussed herein.

There is one crystal structure of human CYP 1A2, PDB code 2HI4 [113], and one of CYP 1B1, PDB code 3PM0 [114]. The structure of both is based on that of the canonical P450 fold – twelve α-helices and four β-sheets, designated A-L and 1–4, respectively – with a few additional helices. Both have the same haem group, protoporphyrin IX, as that in aromatase (Fig. 19). In both enzymes the substrate binding site is a narrow pocket that tightly encloses a molecule of α-naphthoflavone, with many of the residues forming the binding sites being the same in the two proteins. In the following listing of these residues the numbers for CYP 1A2 are given first, with the numbers for the equivalent residues in CYP 1B1 in brackets. One face of the ligand is bordered by I117 (V126), T118 (S127), S122 (S131), T124 (A133), F125 (F134), T223 (N228), F226 (F231), V227 (G232), F256 (L264), N257 (N265), L382 (V395), T385 (T398), I386 (I399), L497 (L509) and T498 (T510). F226 (F231) forms a face-to-face π–π stacking interaction with the ligand. The other face of the ligand is contacted by V220 (L225), F260 (F268), N312 (T325), D313 (D326), F315 (F328), G316 (G329), A317 (A330), F319 (Q332), D320 (D333) and T321 (T334).

**Fig. 23:**
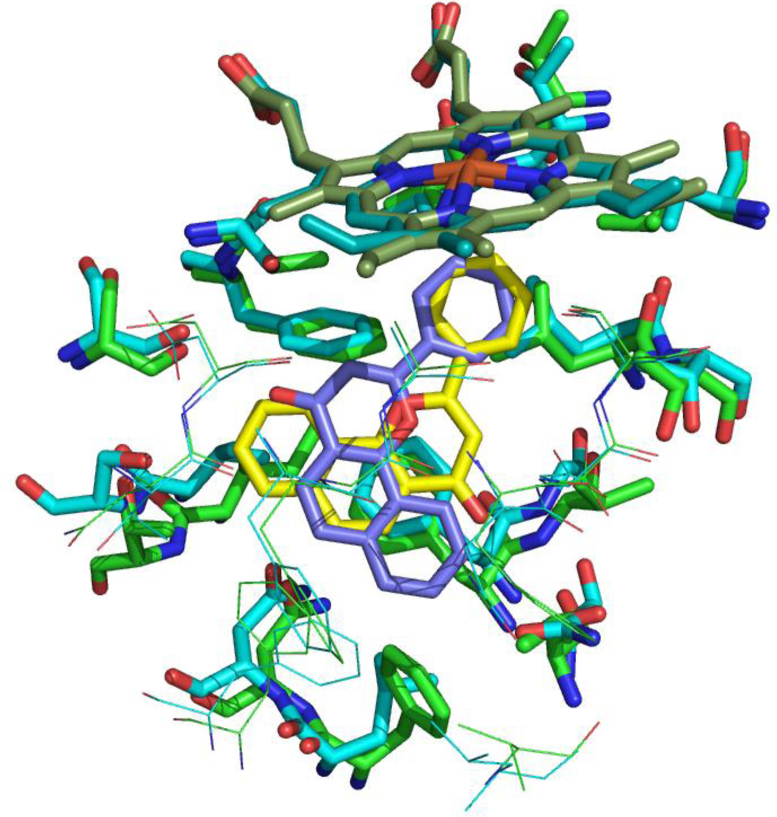
CYP 1A2 and CYP 1B1 substrate binding site structures. Overlay of CYP 1A2 (PDB code 2HI4[113]) and CYP 1B1 (PDB code 3PM0[114]) demonstrating the similarity of the residues forming the substrate binding site and the different pose of the bound ligand, α-naphthoflavone. To avoid obscuring the ligand residues in the foreground are shown as lines rather than sticks. CYP 1A2 – green protein/haem, yellow ligand. CYP 1B1 – blue protein/haem, purple ligand. (For interpretation of the references to colour in this figure legend, the reader is referred to the web version of this article.)

Since both enzymes can hydroxylate estrone at either the 2- or 4-position there must be sufficient flexibility in the substrate binding site, despite the restrictiveness observed in the crystal structures, to allow the substrate to bind in different poses to present either the 2- or 4-positions to the catalytic centre. That such flexibility of substrate binding exists is demonstrated by the fact that the pose of the α-naphthoflavone in one structure is the opposite way round to that in the other structure (Fig. 23).

**Fig. 24:**
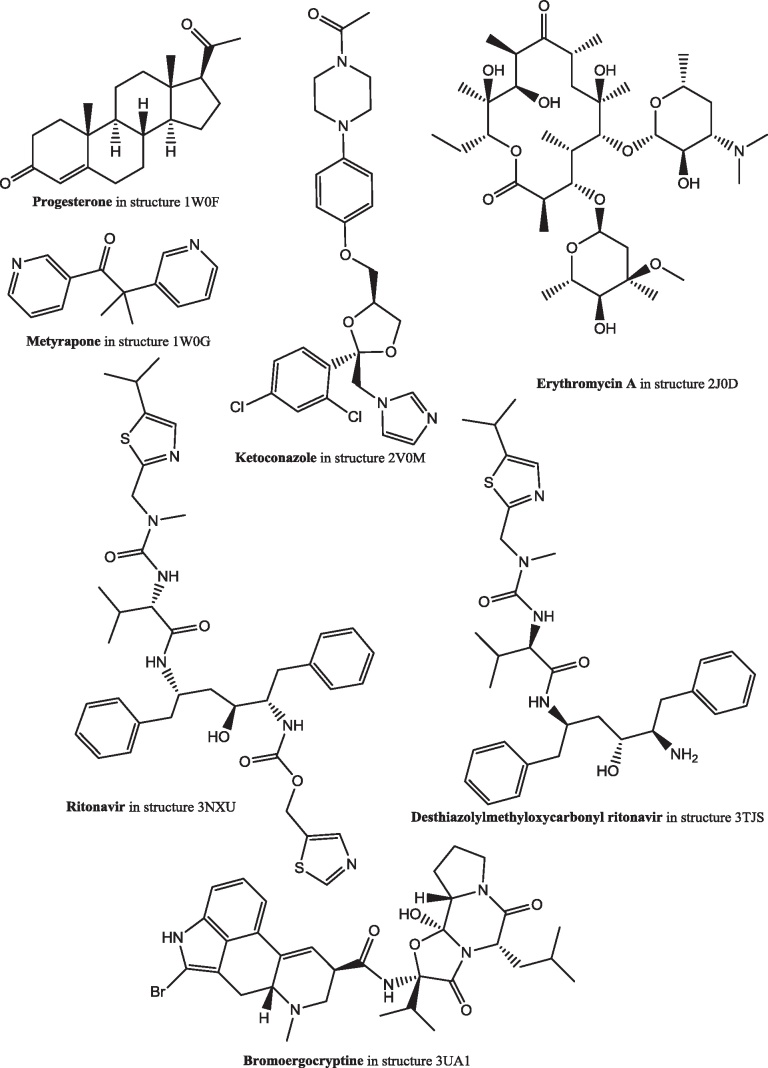
The structures of the ligands in the crystal structures of cytochrome P450 3A4.

**Fig. 25:**
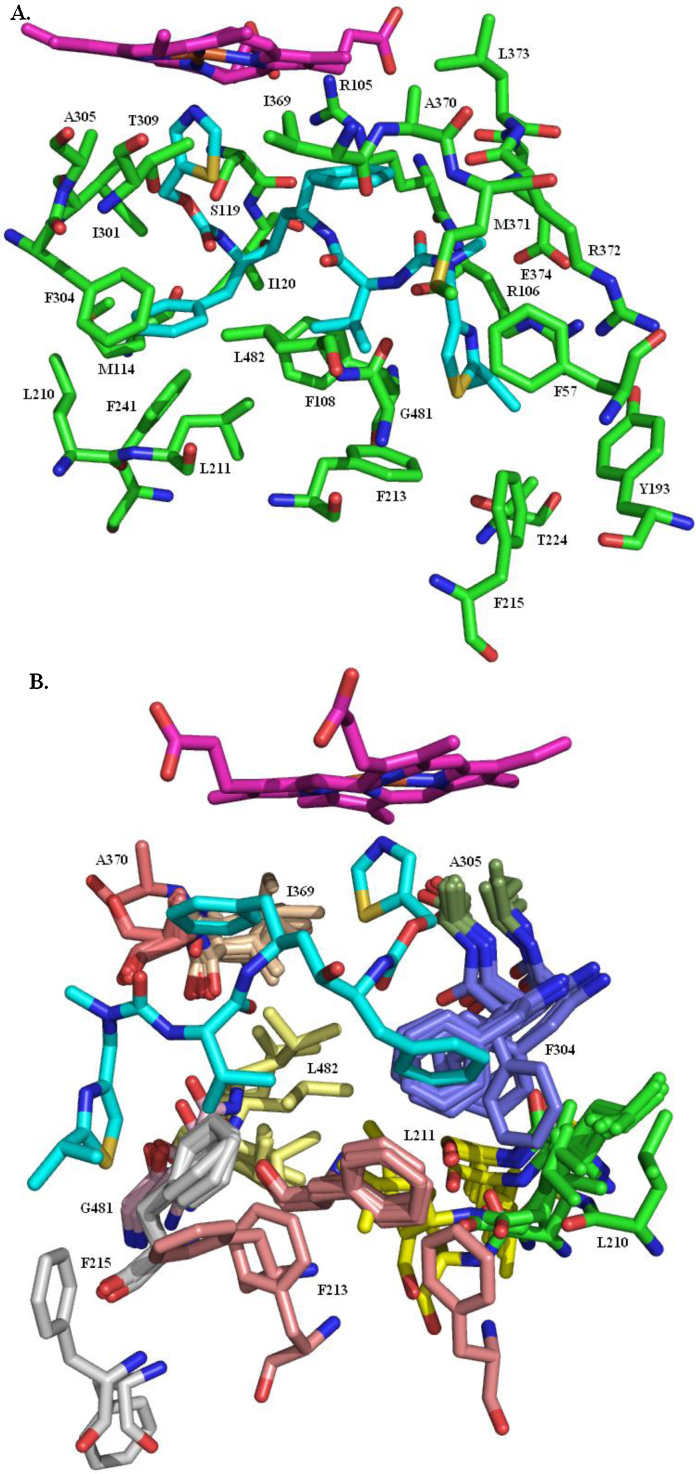
The CYP450 3A4 substrate binding site. (A) Ritonavir in the substrate binding site of CYP450 3A4. Taken from the 3NXU crystal structure [118]. (B) The variability in the shape of the substrate binding site. All nine structures are superimposed and equivalent residues coloured the same. The haem and ritonavir from the 3NXU structure are shown. In both figures the haem is shown in purple and the ritonavir in cyan. (For interpretation of the references to colour in this figure legend, the reader is referred to the web version of this article.)

There are nine publicly available structures of CYP 3A4 (Table 3). This enzyme catalyzes hydroxylation at the 16α-position. In the various structures there are seven different structurally diverse ligands (Fig. 24), though one of these, progesterone, is not in the substrate binding site but in a groove on the surface. To accommodate these ligands there is considerable variability in the shape of the substrate binding site (Fig. 25). This variability shows that there is plenty of space to accommodate estrone such that it presents the 16-position for hydroxylation.

## 8 Catechol *O*-methyl transferase (COMT)

The 2- and 4-hydroxy groups on the oestrogen A ring can be converted to methoxy groups by catechol *O*-methyl transferase (COMT; EC 2.1.1.6; reviewed in [121]) though it should be noted that hydroxylation and ketonisation of estrogens is not limited to these positions: Zhu and Conney [122] list hydroxylations at the 1-, 2-, 4-, 6-, 7-, 11-, 14-, 15-, 16-, 17- and 18-positions. This enzyme is regarded as a phase II drug metabolising enzyme that catalyzes a detoxifying step in drug metabolism [123]. Hydroxylation and methylation are the first steps in the oestrogen degradative and excretory pathways, though the hydroxylated and methoxylated compounds may have roles beyond being mere waste products: see, *e.g.*, [124].

**Fig. 26:**
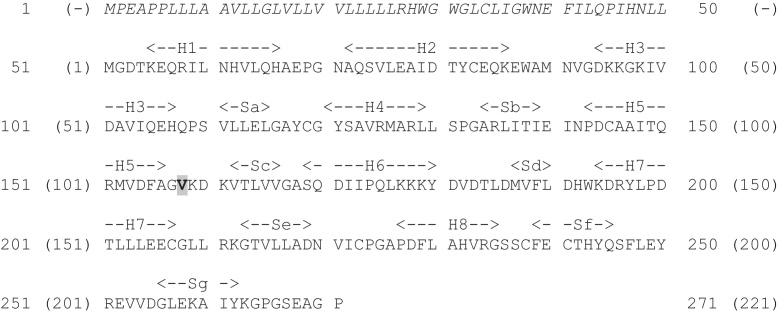
The sequence of human COMT. UniProt ID code P21964. The fifty residues present in mb-COMT but absent in s-COMT are shown in italics. The residue numbering, 1-271, is for mb-COMT: s-COMT is numbered 1–221 (shown in brackets) starting from residue 51 of mb-COMT. Highlighted is residue 158 (mb-COMT) or 108 (s-COMT) that is the site of a V/M polymorphism. Secondary structure elements are labelled: H – helix; S – sheet.

COMT exists in two forms, soluble (s-COMT, 221 residues) and membrane bound (mb-COMT, 271 residues) that differ only in the presence of an additional fifty residues at the N-terminal end of the membrane-bound form (Fig. 26). The residue numbering for s-COMT, 1–221, will be used herein. Residue 108 is a site of a common polymorphism: it can be either valine or methionine. The rarer methionine polymorphism has been associated with a variety of neuropsychiatric disorders [125], increased risk of breast cancer [126,127], lower pain threshold [128] and improved cognitive function involving working memory [129]. These different effects of the polymorphs may be due to the slightly greater enzyme activity of the valine variant [130–132] and/or the greater range of conformational states of the methionine variant observed in molecular dynamics simulations [133] that were suggested by the greater instability (with respect to temperature, autoxidation and denaturants) of the methionine variant (see, *e.g.*, [134] and references therein).

**Fig. 27:**
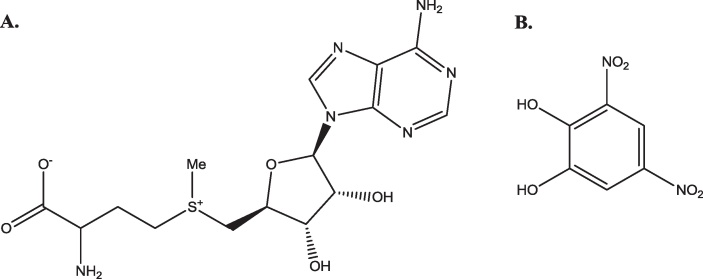
Ligands in the crystal structures of COMT. (A) The structure of S-adenosyl methionine (SAM). (B) 3,5-dinitrocatechol (DNC).

**Fig. 28:**
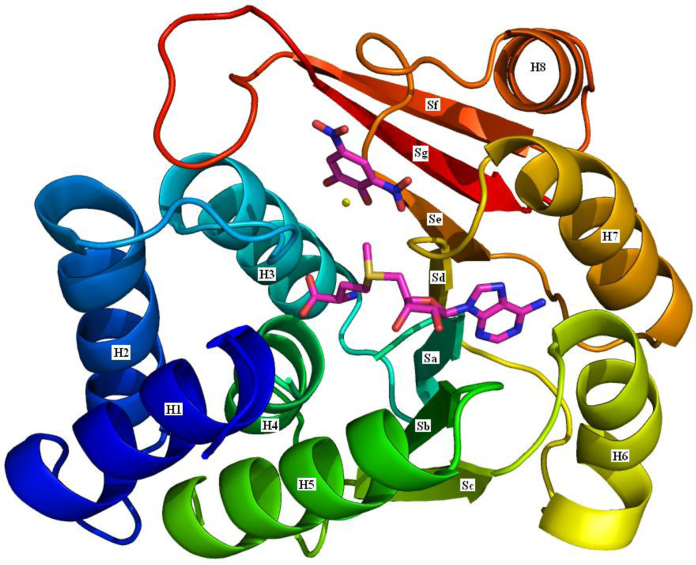
The structure of s-COMT. A cartoon of the s-COMT structure coloured blue at the N-terminus through to red at the C-terminus. The secondary structure elements are labelled as in Fig. 27: H – helix and S – sheet. SAM and DNC are shown with purple carbons. The yellow sphere is a magnesium ion. Based on the 3BWM structure [136]. (For interpretation of the references to colour in this figure legend, the reader is referred to the web version of this article.)

There are three crystal structures of human s-COMT, one of which is of the methionine variant (3BWY; [135]) and the others of the valine variant (3BWM; [136]: 3A7E; unpublished). All three have the methyl group donor, *S*-adenosyl methionine (SAM), and an inhibitor, 3,5-dinitrocatechol (DNC), bound (Fig. 27). The structure of s-COMT is one of two sets of α-helices sandwiching a seven-stranded β-sheet core (Fig. 28). A shallow cleft on the protein surface is occupied by SAM, DNC and a magnesium ion. The SAM binding pocket has a base formed by the C-terminal ends of β-sheets a–d with the walls of the pocket formed by helix 6 and the loops between helix 2 and helix 3, β-sheet a and helix 4, and β-sheet d and helix 7. The SAM methyl group is directed towards the substrate binding site and the catechol oxygen to be methylated.

**Fig. 29:**
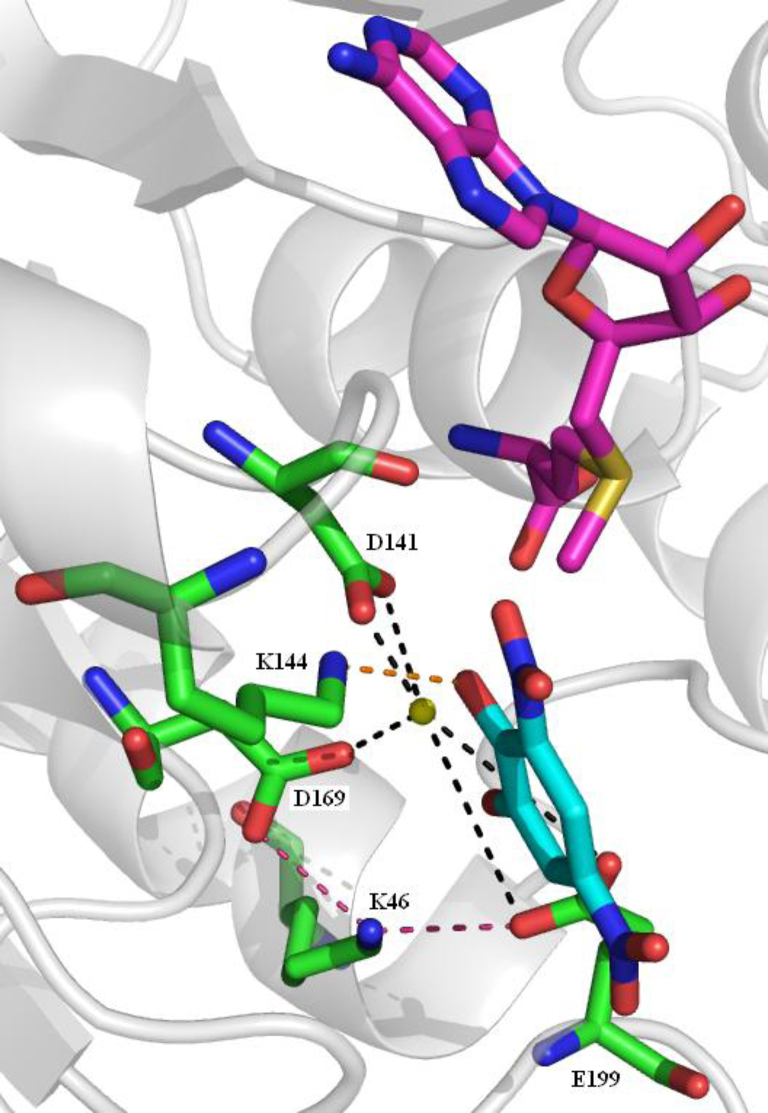
The COMT substrate binding site. SAM is shown with purple carbons, DNC with cyan carbons. Five amino acid side chains are shown with green carbons. The interaction of K144 with a potential substrate is shown by the orange dashed line. Interactions between three acid groups (D141, D169 and E199) and the metal ion are shown by the black dashed lines. The interactions of D169 and E199 with K46 are shown by the pink dashed lines. (For interpretation of the references to colour in this figure legend, the reader is referred to the web version of this article.)

The methylation reaction is dependent on an initial strict sequential order of reactant binding. SAM binding is followed by the binding of a magnesium ion which is followed by the catechol entering the binding site [130,136]. This order is necessary because the binding of one changes the shape of the binding site so that the next can bind. If reactant binding does not occur in this order a dead-end complex is formed. The magnesium ion is held in place by coordination with three acidic residues, D141, D169 and E199 the latter two of which are oriented by interaction with K46 (Fig. 29). The oxygen acting as the methyl recipient is activated by K144 which acts as a general base in the methylation. In a direct bimolecular transfer the SAM methyl group is transferred from the sulphur to the catechol hydroxyl oxygen through an S_N_2-like transition state [137].

COMT inhibitors have been developed primarily for the treatment of Parkinson's disease [138,139].

## 9 Discussion

**Fig. 30:**
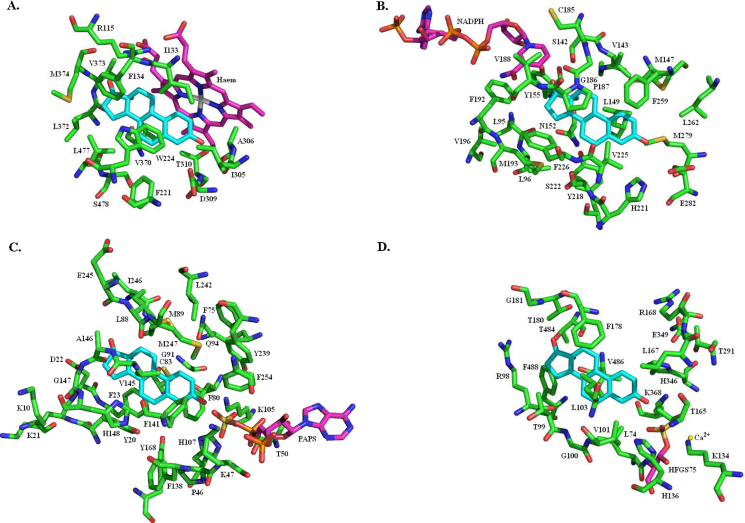
Structure comparison of four oestrogen binding proteins. Shown with estrone (cyan) in the substrate binding site and in the same orientation: (A) aromatase (haem in purple); (B) 17β-HSD1 (NADP in purple); (C) sulfotransferase (PAPS in purple); (D) sulfatase (HFGS75 in purple). (For interpretation of the references to colour in this figure legend, the reader is referred to the web version of this article.)

**Fig. 31:**
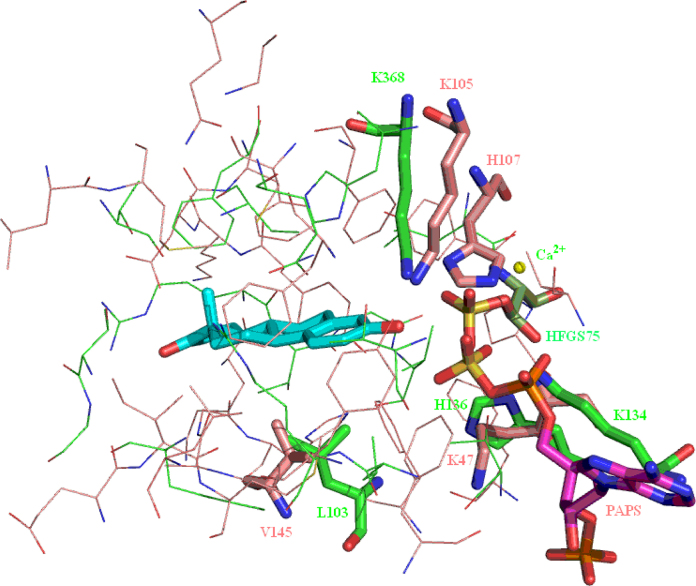
Overlay of the sulfotransferase and sulfatase oestrogen binding sites. Comparison of sulfotransferase (protein: pink carbons, PAPS: purple carbons) and sulfatase (protein: light green carbons, HFGS75: dark green carbons). Estrone is shown with cyan carbons. (For interpretation of the references to colour in this figure legend, the reader is referred to the web version of this article.)

Sulfotransferase, sulfatase, 17β-HSD1 and aromatase all have to recognise estrone (as either a substrate or a product of the catalysed reaction) and at least one other steroid. Despite this common recognition requirement there is no structural similarity between the four proteins in terms of the secondary and tertiary structure and little similarity in the structure of the oestrogen binding sites (Fig. 30). The possible exception to this assertion is, perhaps unsurprisingly given that they catalyse opposite reactions, sulfatase and sulfotransferase. These two enzymes both have a flat surface against which the substrate A and B rings can nestle. This flat surface is formed by L103 in sulfatase and V145 in sulfotransferase (Fig. 31). Both enzymes also have a histidine and two lysines adjacent to the cofactor sulfate group – H136, K134 and K368 in sulfatase, and H107, K47 and K105 in sulfotransferase – possibly suggesting some degree of similarity in the mechanism of the sulfate transfer reactions.

Since estrogens have an important role to play in hormone-dependent cancers the inhibition of the enzymes responsible for producing estrogens is equally important. Historically, most effort has been focused on the development of aromatase and sulfatase inhibitors [140], but more recently considerable effort has been put into the development of 17β-HSD1 inhibitors [87–89]. Aromatase is the only enzyme capable of aromatising the ‘A’ ring thus converting androgens to estrogens. The inhibition of aromatase will, therefore, prevent oestrogen production. However, stores of estrone sulfate (accumulated from the circulation throughout the body at higher levels in breast cancer tissue than normal tissue [141,142]) can be converted to estrone by sulfatase and then to estradiol by 17β-HSD1. With levels of sulfatase and 17β-HSD1 being higher in cancerous tissue than non-cancerous tissue this has been reported to be the main route of oestrogen production in cancerous breast tissue [143–145; see, also, 146] which might suggest that the inhibition of aromatase alone would be unlikely to be a successful treatment in more than a minority of cases. However, aromatase inhibitors such as letrozole, anastrozole and exemestane have proven to be highly effective treatments for many breast cancers [106].

The different binding site structures of the various oestrogen metabolising enzymes mean that specific inhibitors can be designed. A more recent approach, however, is to design molecules that inhibit multiple drug targets, and the first reports of single molecule dual aromatase-sulfatase inhibitors have been published [147–149]. Inhibitors of other combinations of enzymes are feasible and the development of these will be facilitated by structure-based design. Inhibition of aromatase already reduces circulating levels of estradiol to below detectable levels. The concurrent inhibition of sulfatase and also, perhaps, 17β-HSD1 should cut the localised *in situ* production of estradiol in tumour tissue. By inhibiting aromatase, sulfatase and 17β-HSD1 all oestrogen production should be stopped. This may, however, have adverse consequences as estrogens are known to have protective effects on the heart [150]: the incidence of cardiovascular disease in postmenopausal women is three times that of premenopausal women of the same age [151]. This is due, at least in part, to the increased level of aldosterone [152] which is synthesised by CYP11B2 (aldosterone synthase). The simultaneous inhibition of CYP19 (aromatase) and CYP11B2 should reduce the levels of both oestrogen and aldosterone thus having the desired anti-cancer and anti-cardiovascular disease effects. The first dual inhibitors of CYP19 and CYP11B2 have recently been reported [8].

## Figures and Tables

**Table 1:** The crystal structures of sulfotransferases. The mutations are all remote from the substrate and cofactor binding sites and are not expected to have any influence on binding or catalysis.

Protein	PDB code	Ligands	Mutations	Reference
Human 1E1	1HY3	PAPS	V269E	[38]
	1G3M	PAP, polychlorinated biphenol	–	[39]

Murine	1AQU	PAP, estradiol	–	[40]
	1AQY	PAP	–	[40]
	1BO6	PAP, vanadate	–	[41]

Human 1A1	1LS6	PAP, *p*-nitrophenol	–	[46]
	1Z28	PAP	–	[45]
	2D06	PAP, estradiol	–	[44]
	3U3J	PAP	R213H, V223M	[47]
	3U3K	PAP, naphthalene-2-ol	R213H, V223M	[47]
	3U3M	PAP, 3-cyano-7-hydroxycoumarin	R213H, V223M	[47]
	3U3O	PAP, 3-cyano-7-hydroxycoumarin	R213H, V223M	[47]
	3U3R	PAP, *p*-nitrophenol	R213H, V223M, D249G	[47]

Human 1A3	1CJM	Sulphate	–	[42]
	2A3R	PAP, dopamine	–	[43]

**Table 2:** The crystal structures of 17β-HSD1. No papers describing the 3DEY, 3KLP and 3KM0 structures have been published. The structures of the ligands are shown in Fig. 14.

PDB code	Mutated residues	Ligands	Reference
1A27	–	NADP, estradiol	[69]
1BHS	–	–	[70]
1DHT	–	5α-Dihydrotestosterone	[71]
1EQU	–	NADP, equilin	[72]
1FDS	–	Estradiol	[73]
1FDT	–	NADP, estradiol	[73]
1FDU	H221L	NADP, estradiol	[74]
1FDV	H221L	NAD	[74]
1FDW	H221Q	Estradiol	[74]
1I5R	–	DHETNA^a^	[75]
1IOL	D112E	Estradiol	[76]
1JTV	–	Testosterone	[77]
1QYV	–	NADP	[78]
1QYW	–	NADP, 5α-androstan-3,17-dione,	[78]
1QYX	–	NADP, 4-androstene-3,17-dione	[78]
3DEY	–	5α-Dihydrotestosterone	–
3DHE	–	3β-Hydroxy-5-androsten-17-one	[71]
3HB4	–	E2B^b^	[79]
3HB5	–	NADP, E2B^b^	[79]
3KLM	–	5α-Dihydrotestosterone	[80]
3KLP	–	(3α,8α,17β)-androst-5-ene-3,17-diol	–
3KM0	–	NADP, 5α-androstan-3β,17β-diol	–

a DHETNA: O5′-[9-(3,17β-dihydroxy-1,3,5(10)-estratrien-16β-yl)-nonanoyl]adenosine.

b E2B: 3-(((8*R*,9*S*,13*S*,14*S*,16*R*,17*S*)-3,17-dihydroxy-13-methyl-7,8,9,11,12,13,14,15,16,17-decahydro-6*H*-cyclopenta[*a*]phenanthren-16-yl)methyl)benzamide.

**Table 3:** The crystal structures of cytochrome P450 3A4. The structures of the ligands are shown in Fig. 22.

PDB code	Ligands	Reference
1TQN	–	[115]
1W0E	–	[116]
1W0F	Progesterone^a^	[116]
1W0G	Metyrapone	[116]
2J0D	Erythromycin A	[117]
2V0M	Ketoconazole^b^	[117]
3NXU	Ritonavir	[118]
3TJS	Desthiazolylmethyloxycarbonyl ritonavir	[119]
3UA1	Bromoergocryptine	[120]

a The progesterone is not in the substrate binding site, but in a groove on the surface of the protein.

b There are two molecules of ketoconazole in the substrate binding site.
